# Magnesium as an Adjuvant to Rocuronium to Improve Intubation Conditions and Shorten the Intubation Time: A Randomized Controlled Study

**DOI:** 10.7759/cureus.68712

**Published:** 2024-09-05

**Authors:** Franklin J Roy, Swetha Ramani, Karthika Urkavalan, Shaheen Khan

**Affiliations:** 1 Anaesthesiology, SRM Medical College Hospital and Research Centre, Chennai, IND

**Keywords:** intubation, magnesium sulphate, pretreatment, priming, rocuronium

## Abstract

Aim and objective: The purpose of the study is to compare the effects of rocuronium priming with the combined technique of magnesium pretreatment and rocuronium priming and to investigate whether this pretreatment could further accelerate the onset of neuromuscular blockade during intubation.

Materials and methods: A double-blinded randomized controlled trial (RCT) clinical study was done on patients at a tertiary care center for six months after obtaining approval from the institutional ethical committee. A total of 150 patients were randomly allocated as Group MP (infusion of 50 mg/kg of MgSo4 over 10 min was given 10 mins prior to premedication and dose of rocuronium 0.06 mg/kg given three minutes), Group P (priming dose of rocuronium 0.06 mg/kg given three minutes before the intubating dose), and Group C (control group with the same volume of 0.9% saline and rocuronium bolus of 0.6 mg/kg on intubation). Parameters such as demographic and hemodynamical data, American Society of Anesthesiologists (ASA) score, Mallampati scoring, neuromuscular monitoring, intubation grading, and number of successful/failed attempts were recorded.

Results: Our results showed that Group MP had a rapid onset of action of rocuronium with 58.90 +/- 4.77 seconds and a longer duration of action of rocuronium with 54.92 +/- 10.39 minutes, which are statistically significant compared to Group P (onset of action of ROC 106.70 +/- 4.24 seconds and duration of action rocuronium 45.88 +/- 6.22 minutes) and Group C (onset of action of ROC 154.56 +/- 11.39 seconds and duration of ROC 40.56 +/- 3.96 minutes). The maximum number of patients in Group MP (33 patients) showed good intubation conditions compared to Group P (23 patients) and Group C (16 patients), which was statistically significant.

Conclusion: We conclude that magnesium sulfate pretreatment in combination with rocuronium priming (Group MP) considerably accelerates the onset of rocuronium action, increases the duration of action of rocuronium, and enhances the intubation procedure without any adverse effect of rocuronium and magnesium sulfate.

## Introduction

Achieving an ideal intubating condition within a short period is the primary goal in any general anesthesia, as this will avoid unnecessary intubation failure and laryngeal or tracheal injury in the perioperative period [1,2]. This is possible by using neuromuscular blocking drugs. An ideal neuromuscular blocking drug should be rapid-acting, short-acting, without any cardiopulmonary adverse effects, metabolized, and eliminated without residual drug effect [3-4].

To our current knowledge, succinylcholine is the only drug that produces rapid onset of neuromuscular blockade and an ideal intubating condition, but it is not without any contraindications and complications. Hyperkalemia; raised intraocular, intraabdominal, intracerebral pressures; and postoperative myalgia are a few [5]. The search for a faster onset neuromuscular blocking drug without any adverse effects is still on. The closest one to succinylcholine is rocuronium at the dosage of 1.2 mg/kg, producing similar onset time and conditions for intubation, but it comes with the cost of a longer duration of action requiring sugammadex for its reversal, which is not easily available in many centers [6].

Several studies were conducted on various techniques to hasten the onset time of nondepolarizing muscle relaxants without increasing the dosage [7-9], of which the priming technique was one such proven technique used in anesthesia practice for many decades. Rocuronium priming shortens the onset time at the standard dosage of 0.6 mg/kg, but the intubating conditions were not always ideal. To search for newer alternatives, additives such as ephedrine, ketamine, and magnesium sulfate were studied, which when added with nondepolarizing muscle relaxants were found to shorten their onset time and simultaneously produce good intubating conditions [7-10].

Magnesium is used extensively in anesthesia nowadays, especially in opioid-free anesthesia [11]. Being one of the abundant cations in the body, it is used in many enzymatic reactions as a cofactor. It has potentiating action on nondepolarizing muscle relaxants by decreasing the release and reducing the effect of acetylcholine in the neuromuscular junction. Its analgesic effect enables the anesthetist to reduce the use of anesthetics and analgesics in the perioperative period. Moreover, its use does not prolong the reversal time [12-15].

This study was conducted to evaluate the intubation conditions and the onset time of neuromuscular blockade when magnesium is used as an additive to rocuronium and compared with that of priming of rocuronium.

## Materials and methods

This study was a prospective and double-blinded randomized controlled study (RCT), conducted over 18 months after getting approval from the SRM Medical College Hospital and Research Center Institutional Ethical Committee (Clinical Trials Registry - India (CTRI), registration number: 2020/05/023399). All adult patients who were admitted to SRM Medical College Hospital and Research Center, a tertiary care hospital in Chennai, India, for surgical procedures under general anesthesia, aged 18-65 years, belonging to American Society of Anaesthesiologists (ASA) grades I and II were included. Randomization was done by computer-generated random numbers kept in a sealed envelope cover.

Excluded from the study were the ones who refused to participate; patients with cardiac disease, chronic kidney disease, chronic liver disease, respiratory disease, pregnancy, and lactation; and patients with a known difficult airway, known allergy to study drugs, and preexisting neuromuscular disorders or neuropathies.

A study was done with 50 patients in each group (a total of 150); the sample size was detected based on the primary outcome, which was the intubation condition in the parent study.

Patients were allocated into three equal study groups, each containing 50 patients, after obtaining written consent, based on the computer-generated random numbers. Both the investigators and the patients were blinded to the study. Three anesthesiologists were involved in the study, of which two of them were unrelated to the study. The drugs were prepared by the first anesthesiologist as per the protocol.; the second anesthesiologist administered the set of medications given to him and monitored the train-of-four (TOF) and recorded the findings. An experienced anesthesiologist intubated the patients using direct laryngoscopy with a Macintosh blade and noted down the intubation score and Cormack-Lehane score. The patients who were assessed earlier were given a premedication tab alprazolam 0.5 mg orally, the night before surgery. On the day of surgery, once the patient was wheeled into the operation room, standard monitors such as noninvasive blood pressure, heart rate, and peripheral O_2_ saturation and electrocardiograph were attached, and baseline parameters were recorded. After securing the appropriately sized intravenous cannula, 100 ml of plain normal saline or magnesium sulfate was infused over 10 minutes, according to the group allocation.

In the magnesium with rocuronium priming group (Group MP), patients were administered an intravenous infusion of 50 mg/kg of magnesium sulfate in 100 ml 0.9% normal saline over 10 minutes. In priming and control groups (Group P and Group C), plain 100 ml of 0.9% normal saline infusion was given over 10 minutes. After premedication with 0.05 mg/kg of midazolam intravenously, neuromuscular monitoring was performed with a TOF monitor (STIMPOD NMS 450 X). On the forearm with no intravenous line or blood pressure cuff, two surface electrodes were placed along the ulnar nerve at 3-5 cm distance apart. The supramaximal current was estimated from the single twitch response monitored every 10 seconds.

A priming dose of rocuronium 0.06 mg/kg (made to 1 ml) was given in Group MP and Group P, whereas 1 ml of 0.9% of normal saline was given in the control group. Inj. fentanyl (2 mcg/kg), inj. propofol (2 mg/kg), and rocuronium (0.54 mg/kg in Group MP and Group P and 0.6 mg/kg in Group C) made to the same volume (5 ml), were given three minutes after the priming dose.

Laryngoscopy and intubation were performed after the disappearance of the single twitch, and the intubating condition as per Cooper’s score [16] was documented by the experienced anesthetist, who is blinded to the groups. The time of disappearance of the single twitch was taken as the onset time of neuromuscular blockade (T1). Laryngoscopy was attempted and the intubation score along with the Cormack-Lehane score was noted. The trachea was intubated with an appropriately sized endotracheal tube. Hemodynamic parameters such as pulse rate, mean arterial pressure, and oxygen saturation were recorded every minute till 10 minutes of intubation. Anesthesia was maintained with controlled ventilation with a 50% nitrous-oxygen mixture with 1% sevoflurane and rocuronium top-ups based on the TOF response. Intraoperatively, temperature was monitored with the nasopharyngeal probe, and body temperature was maintained at ≥35°C.

After noting the onset time, the stimulation was then switched over to a TOF mode. The appearance of two responses in TOF was taken as the duration of neuromuscular blockade (T2). Total intraoperative requirement of rocuronium and adverse effects like bradycardia (20% from baseline) and hypotension (20% from baseline) were recorded. Any complaints such as breathing difficulty, flushing, or generalized weakness were documented. At the end of the procedure, neuromuscular blockade was reversed with inj. sugammadex 4 mg/kg and monitored in the post-anesthesia care for 24 hours.

**Figure 1:**
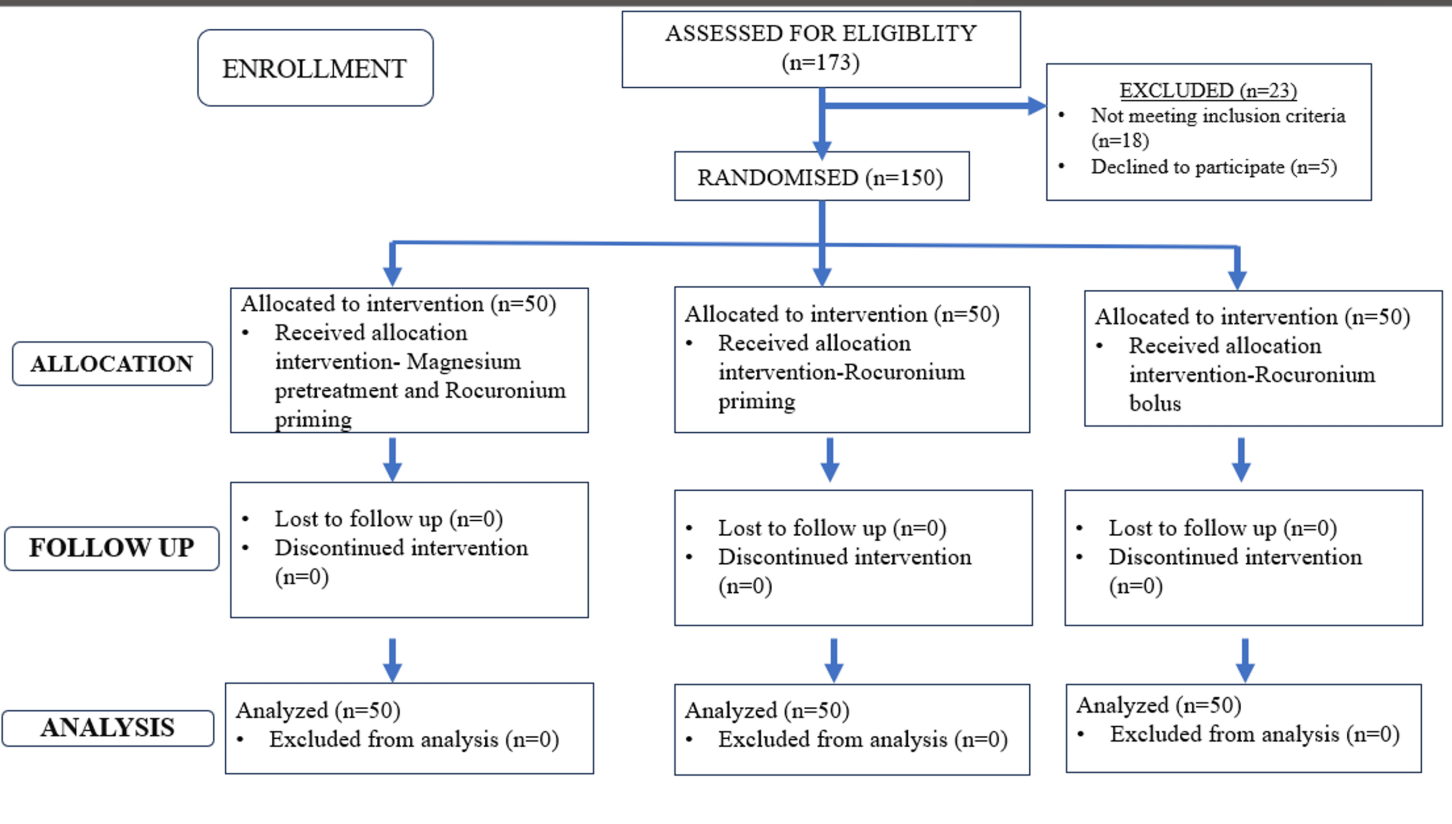
Consolidated Standards of Reporting Trials (CONSORT) diagram

Sample size calculation was done based on Kim et al.'s study, where the onset time for rocuronium priming was 125 +/- 47 seconds. Data were expressed as mean ± standard deviation (SD) or percentages. Statistical analysis was done with IBM SPSS Statistics for Windows, version 23.0 (released 2021, IBM Corp., Armonk, NY). The chi-square test or Fisher's exact test was used for the sex ratio. One-way analysis of variance (ANOVA) for age, weight, and height. P < 0.05 was considered statistically significant. Figure 1 shows the Consolidated Standards of Reporting Trials (CONSORT) diagram.

## Results

There was a normal distribution of all the datasets. There was no difference in the demographic profile among the three groups (Table 1). The baseline hemodynamic parameters - non-invasive blood pressure (NIBP), heart rate (HR), and oxygen saturation (SpO_2_) - showed no difference.

**Table 1:** Demographic profile The demographic data in all three groups are similar, and it was statistically not significant (p-value < 0.05).

Demographic data				
Groups	MP	P	C	Sig
Parameters	Mean ± SD	Mean ± SD	Mean ± SD	P-value
Age	36.98 ± 9.47	40.90 ± 9.55	41.86 ± 11.23	0.081
Weight (kg)	65.20 ± 7.84	59.04 ± 8.73	68.78 ± 7.35	0.079
Height (cm)	164.88 ± 9.87	170.58 ± 6.60	159.08 ± 8.50	0.061
BMI	24.20 ±4.00	23.08 ±3.96	25.82 ±4.29	0.073

**Figure 2:**
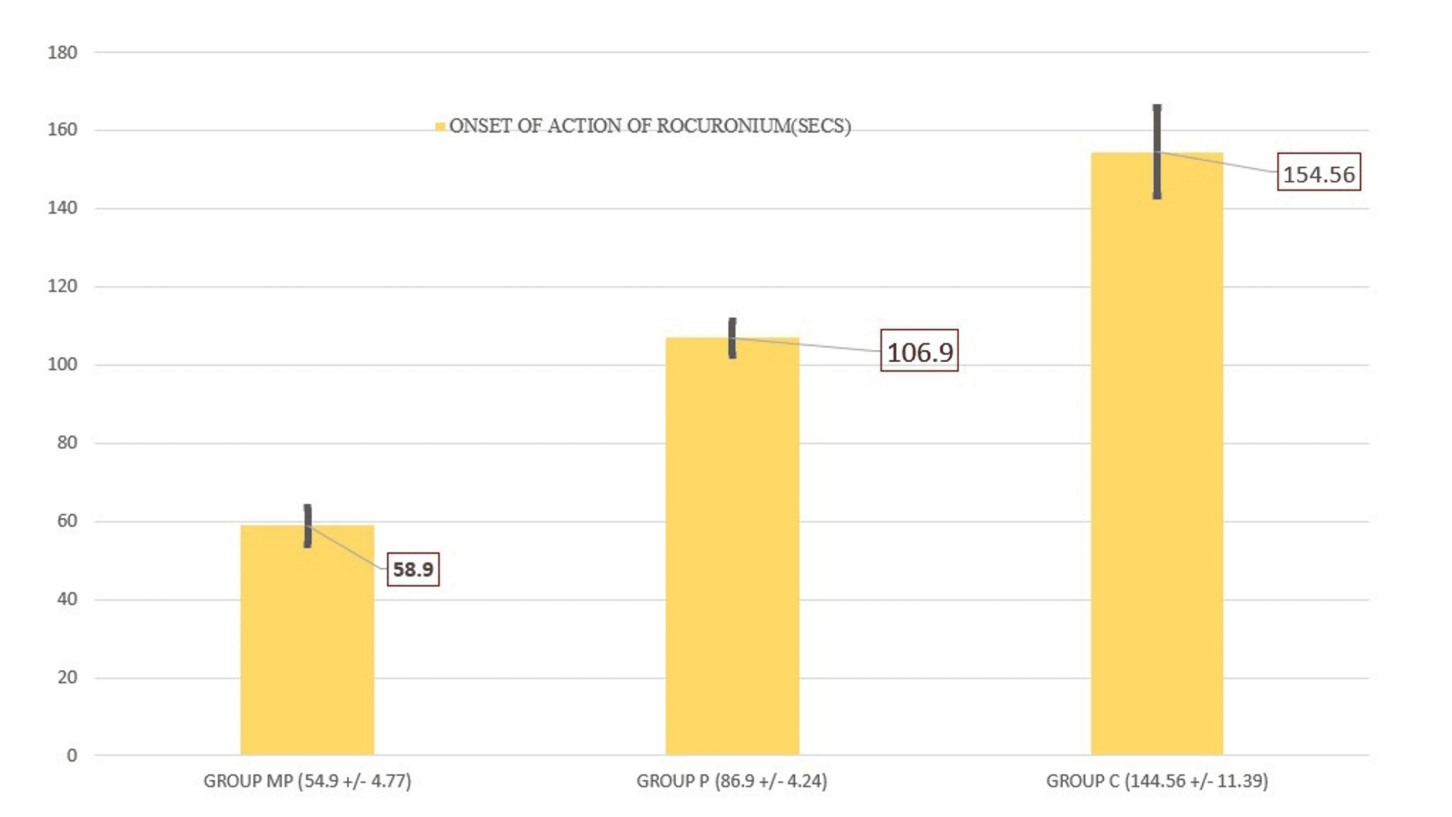
Onset of action of rocuronium

**Figure 3:**
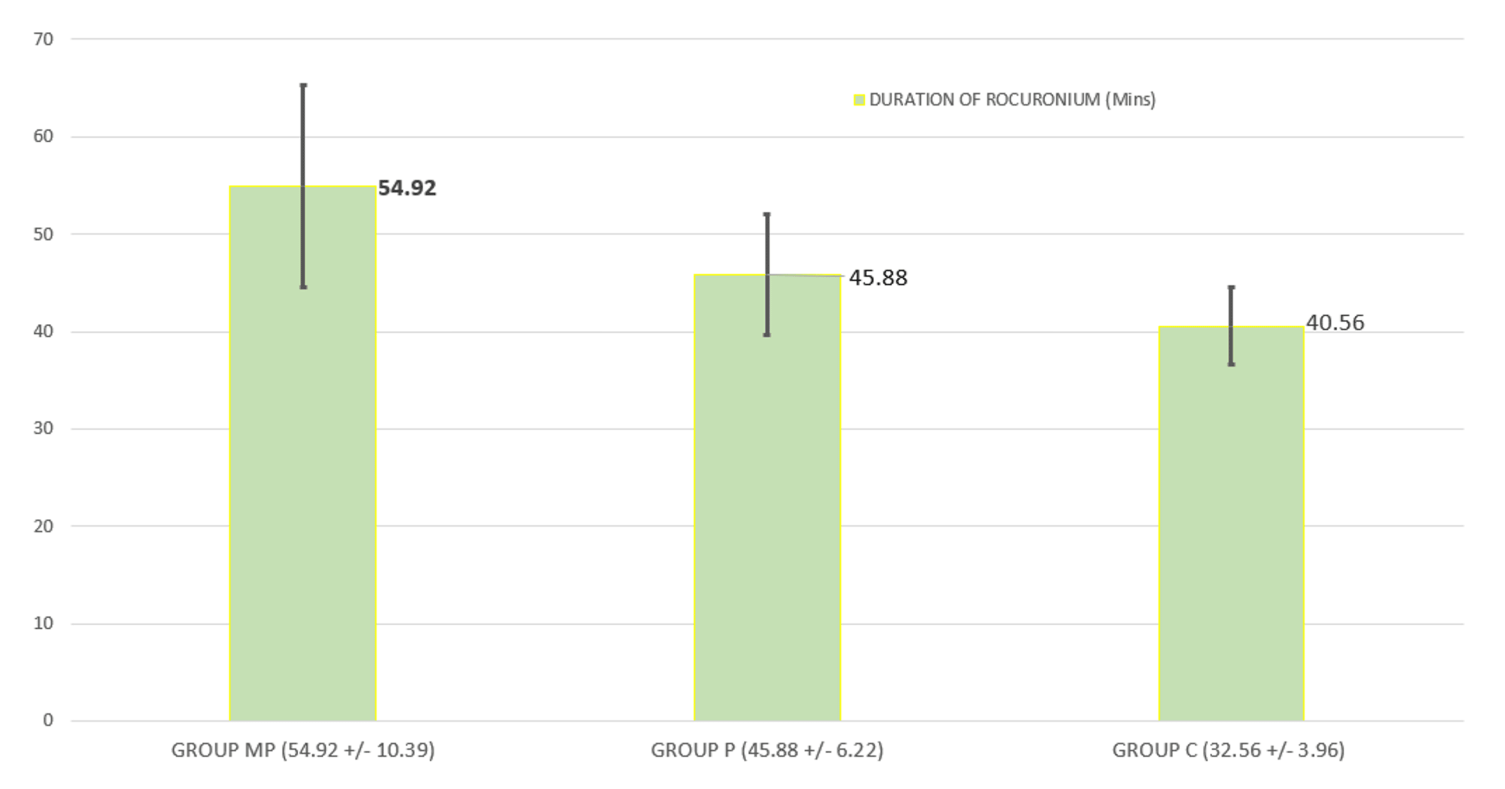
Duration of action of rocuronium

The results showed that Group MP had a rapid onset of action of rocuronium with 58.90 ± 4.77 seconds (Figure 2) and a longer duration of action of rocuronium with 54.92 ± 10.39 minutes (Figure 3), which were statistically significant when compared to Group P (onset time was 106.70 ± 4.24 seconds and the duration of action of rocuronium was 45.88 ± 6.22 minutes) and Group C (onset time was 154.56 ± 11.39 seconds and the duration of rocuronium 40.56 ± 3.96 minutes) (p = 0.0001).

**Figure 4:**
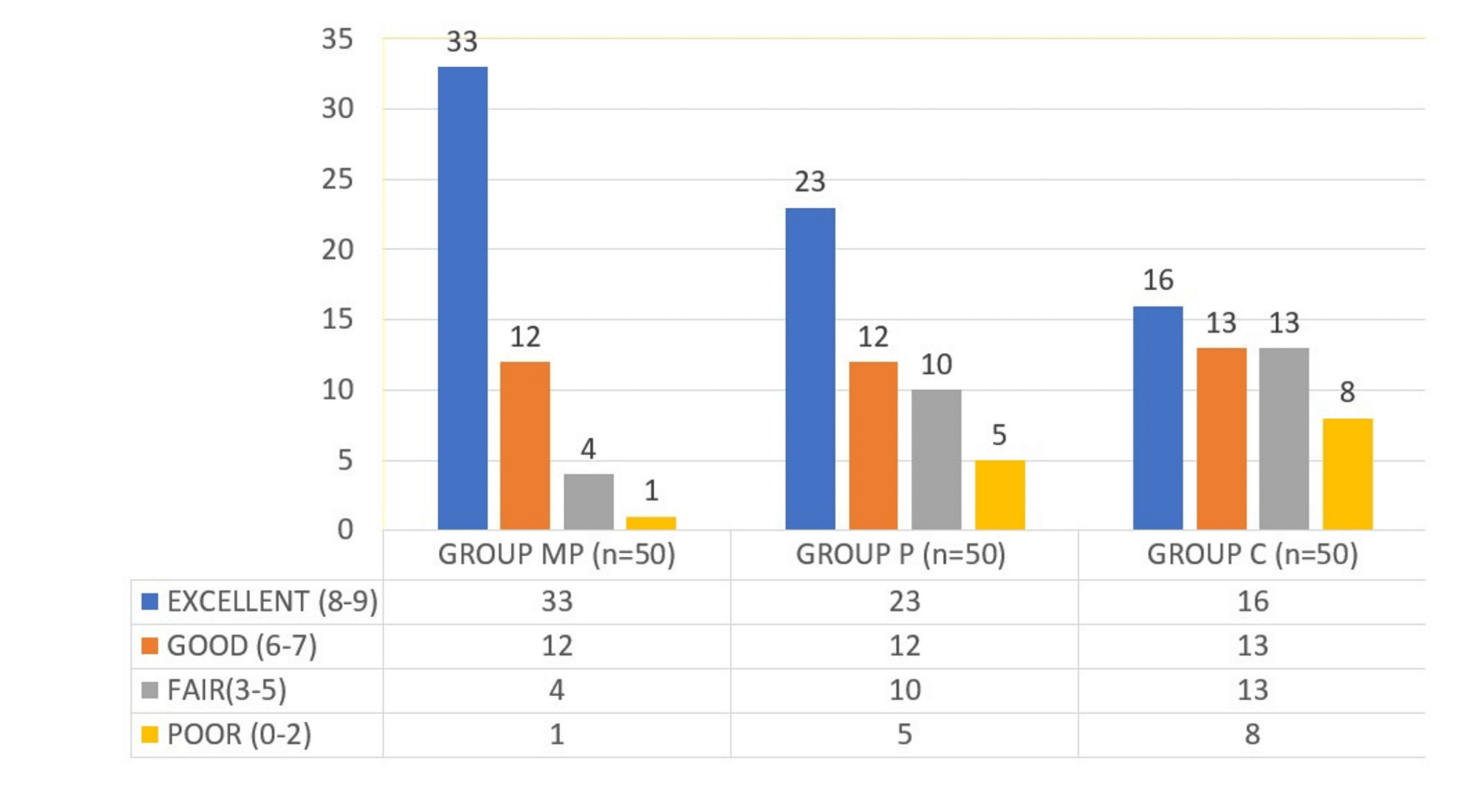
Intubation score

The maximum number of patients in Group MP (33 patients) showed the best intubation conditions compared to Group P (23 patients) and Group C (16 patients), which was statistically significant (p = 0.0001). Tracheal intubation was successful in all the patients, but the intubation score was poor in eight patients in Group C (Figure 4).

**Figure 5:**
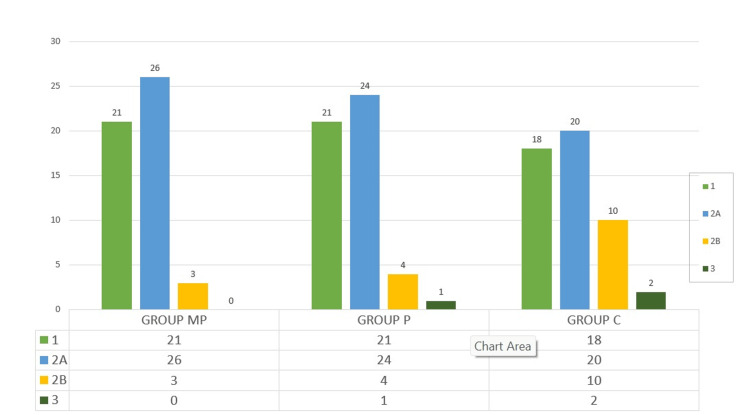
Laryngoscope view

The laryngoscope view, Cormack-Lehane score of 1, was seen in Group MP (21 cases) and Group P (21 cases) than when compared with Group C (18 cases) (Figure 5).

Seven and 11 patients complained of difficulty breathing in Group MP (14%) and Group P (22%), respectively, while administering the priming dosage of rocuronium. A sensation of warmth and flushing was observed in eight patients in Group MP (16%) during magnesium infusion. None of the patients showed difficulty in breathing or sensation of warmth and flushing in the control group. No incidence of hypotension or bradycardia was noted in any of the three groups.

## Discussion

The onset time of rocuronium was the shortest in the magnesium and priming group (58.90 ± 4.77 seconds) than the priming-only group (106.90 ± 4.20 seconds), and it was last short in the control group (154.56 ± 11.39 seconds). When compared to the control group, the onset of rocuronium was shortened by 38% and 68% with the priming-only group and magnesium with the priming group, respectively. This result is like the previous study done by Kim et al. [17], which showed 56 ±16 seconds in pretreatment with magnesium sulfate with rocuronium priming, 125 ± 47 seconds for the rocuronium priming group, and 150 +/- 56 seconds for rocuronium bolus.

The priming technique is one when 10% of the intubating dose of the neuromuscular blocking agent is given followed by the remaining 90% of the dose after three minutes [18]. This ensures adequate intubating conditions within a short time, neither prolonging the duration of the neuromuscular blocking drug nor any untoward side effects. The priming dose is supposed to occupy the acetylcholine receptors and reduce the time for the remaining drug to act. The priming dose also blocks the presynaptic receptors and reduces the release of acetylcholine, facilitating the intubation dose to act faster and more effectively. As important as the dose, the timing of priming also plays a major role in the onset time. For rocuronium, it is three minutes, whereas for vecuronium and atracurium, it is more than five minutes [19,20]. We used rocuronium in this study and kept the priming time to three minutes.

Magnesium acts as an antagonist at the N-methyl-D-aspartate (NMDA) receptor and sympathetic reception of the central nervous system, producing both anesthetic and analgesic effects and reducing stress response by decreasing the release of circulating catecholamines. Hence, it is the most widely used adjuvant in anesthesia in recent practice. At the motor end plate, it reduces the release of acetylcholine, enhancing the effectiveness of neuromuscular blocking drugs, and leading to faster onset time with good intubating conditions [13,21-23]. In this study, we found magnesium as an adjuvant to rocuronium, producing profound neuromuscular blockade within a short time.

Adding magnesium as an adjuvant along with the priming technique shortens the onset time of rocuronium without any adverse effects when used in adjuvant dosage. Although the mechanisms of priming and magnesium are different, both together produce adequate intubating conditions.

The duration of rocuronium is longer when magnesium is used as an additive due to its central NMDA receptor antagonistic action, hence reducing the intraoperative requirement of anesthetic agents. The total requirement of rocuronium in this study was less in the magnesium adjuvant priming group when compared with the priming-only group and control. Moreover, in this study, the hemodynamic parameters remained without major change during laryngoscopy and intubation, and throughout the procedure, neither did we observe any bradycardia or hypotension in the magnesium adjuvant priming group.

There are multiple sites to monitor the neuromuscular blockade in which different nerves are stimulated to find the response. The commonest one is the ulnar nerve stimulation, and the response will be observed in the adductor pollicis muscle [24,25]. The adductor pollicis is a better muscle to monitor than the corrugator supercili and orbicularis oculi as per the European Society of Anaesthesiology and Intensive Care (ESAIC) and ASA recent guidelines [26]. In this study, we used the adductor muscle to monitor the onset time of neuromuscular blockade. We used single twitch stimulation to identify the onset time as it the a useful indicator of onset time. As a single twitch is not a good indicator to monitor the duration of neuromuscular blockade, we switched to a train of four stimulations.

Flushing and warmth sensation when magnesium is infused, and it is self-limiting without requiring any other treatment.

Magnesium does not interfere with the reversal time of the sugammadex [27]. In our study, we used 4 mg/kg of sugammadex after obtaining a 0.9 ratio in the train of four in all three groups. We found that the recovery time ranged from 122 seconds, 117 seconds, and 110 seconds in the magnesium adjuvant priming group, priming alone group, and control group, respectively.

Limitation

The non-availability of sugammadex in many centers is one of the limitations of using rocuronium. Although rocuronium and magnesium provide ideal intubation conditions, the cost-effectiveness of using rocuronium, magnesium, and sugammadex for reversal should also be considered. The methods or drugs to alleviate the adverse effects of priming and magnesium during the pretreatment of rocuronium are yet to be studied.

## Conclusions

There is compelling evidence now to suggest that prior administration of magnesium sulphate reduces the requirement of rocuronium intraoperatively. Priming with magnesium in patients receiving rocuronium has evidently demonstrated better intubating conditions, thus increasing the chances of successful intubation. Magnesium sulfate also has no significant disadvantages or major side effects, which makes its use more appealing. An added advantage of using rocuronium is that it can be reversed completely with sugammadex, making it an ideal agent for both rapid sequence induction and intraoperative maintenance of neuromuscular blockade.
